# The 7 Pillars of Multivessel Minimally Invasive Coronary Surgery

**DOI:** 10.1177/15569845211007835

**Published:** 2021-06-08

**Authors:** Saqib H. Qureshi, Marc Ruel

**Affiliations:** 1 27339 Division of Cardiac Surgery, University of Ottawa Heart Institute, ON, Canada

## Introduction

Minimally invasive coronary surgery (MICS CABG) is a robust technique that achieves complete surgical myocardial revascularization while avoiding sternotomy. It has been proven to provide excellent safety and graft patency.^1,2^ With MICS CABG, standard left internal thoracic artery (LITA) plus saphenous vein grafting or multiarterial grafting can be accomplished. Once the learning curve is overcome,^3^ the benefits transferred to patients are multiple and include shorter recovery, less pain, and less morbidity.^4^ Our center has widely reported on our MICS CABG experience,^1,4^ with excellent results. We are honored to share what, in our view, constitutes the 7 pillars of successful MICS CABG.

## 1. The Key is Patient Selection

Patients should be deemed appropriate for surgical myocardial revascularization as per local Heart Team decision, and MICS CABG should be performed at centers with minimally invasive expertise. Prior off-pump CABG expertise is a must. Patients with large body habitus, porcelain aorta, calcified femoral vessels negating potential extracorporeal support, left-sided chest trauma, or previous radiation are not suitable candidates, particularly early in the experience.

## 2. The MICS CABG Team

An anesthetist experienced in off-pump coronary surgery and effective communication between the anesthetist and surgeon are 2 vital attributes to support the flow of the operation. Several anesthetic considerations are cornerstones of the operation such as single-lung ventilation either with the use of a double-lumen tube or single-lumen tube and a bronchial blocker, intravascular volume management to curtail right ventricular expansion, and attentive hemodynamic support during posterior descending artery or left circumflex coronary artery grafting. Transesophageal echocardiography is instrumental to assist cannulation and assess the degree of wall motion abnormalities.

## 3. Setting Up and Gaining Access

**Fig. 1:**
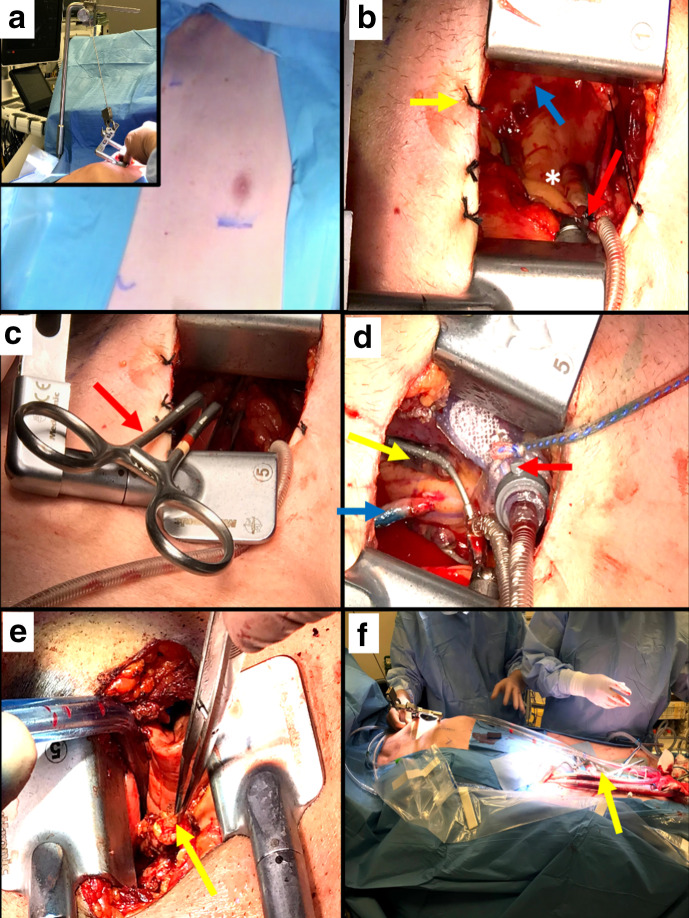
(**a**) Initial positioning of incision and (inset) contralateral traction from patient’s right shoulder for better exposure of the ascending aorta for proximal anastomoses. (**b**) Maneuvers to mobilize ascending aorta into the left chest; yellow arrow: pericardial traction stitches, red arrow: Octopus Nuvo Tissue Stabilizer (Medtronic, Minneapolis, MN, USA), blue arrow: ascending aorta, (*) right ventricular outflow tract. (**c**) Application of side-biting clamp (red arrow) on ascending aorta. (**d**) Positioning and stabilization for distal anastomosis to the posterior descending artery; yellow arrow: Octopus Nuvo Tissue Stabilizer, red arrow: Starfish Evo Heart Positioner (Medtronic, Minneapolis, MN, USA), blue arrow: vein graft to posterior descending artery anastomosis. (**e**) Confirmation of lie of left internal thoracic artery graft under the cover of right lung upon reinflation. (**f**) Cardiopulmonary bypass support during minimally invasive coronary surgery via right groin (yellow arrow).

While the greater saphenous vein can be harvested concurrent to LITA takedown, harvesting of the left radial artery is usually performed prior to final positioning of the patient. The patient is then repositioned in a semi-right lateral decubitus position with the left arm extended, avoiding any traction to prevent brachial plexus injury. Defibrillation pads are placed on the right chest and left back, and surface landmarks are carried out from the sternal angle and xiphisternum. Triangulation is carried out to the fourth or fifth intercostal space in the midclavicular line, for a 5-cm incision (Fig. 1a). After accessing the pleural space, the cardiac apex is palpated and usually should lie one space caudal to the incision. Surgeons should be flexible if needing to change the intercostal space to one space above or below, all through the same skin incision.

## 4. LITA Harvest

With the aid of a Rultract retractor (Rultract, Cleveland, OH, USA), ribs are lifted superiorly and to the left. If the LITA is not immediately seen, separation of medial pericardial reflection from behind the sternum can aid visualization. Harvest can proceed in either skeletonized or pedicled fashion. Before division of the LITA, heparin is given to achieve a target activated clotting time above 280s for an off-pump strategy. Once the LITA is harvested, methylene blue is applied to maintain orientation.

## 5. Right to Left “Relocation” of Ascending Aorta for Proximal Anastomoses

The aortic mobilization is achieved in several incremental steps. Rightward chest wall traction is carried out from the patient’s right shoulder (Fig. 1a, inset image). Multiple pericardial traction stitches are placed (Fig. 1b). Packing is added to the right of the aorta, anterior to the superior vena cava. The Octopus Nuvo Tissue Stabilizer (Medtronic, Minneapolis, MN, USA) is placed on the right ventricular outflow tract, which is gently compressed and pulled downward and to the left to achieve unhindered access to the aorta. Before application of a Kay-Lambert side-biting clamp (Fig. 1c), systolic pressure is brought down to 80 to 85 mm Hg. Proximal anastomoses are performed in a routine fashion and may require a knot pusher for tying.

## 6. Positioning for Stability and Exposure

The sequence of performing distal anastomoses is dictated by surgeon preference, relative ischemia of each territory, and ease of access. Prior to lifting the heart within the closed chest, it is desirable to bring the systolic blood pressure to a range of 130 to 140 mm Hg. To expose the vessels on the inferior surface, the heart is suspended toward the left shoulder and for the lateral surface toward the right hip, by using nonsternotomy stabilizers (Octopus Nuvo Tissue Stabilizer and Starfish Evo Heart Positioner, Medtronic, Minneapolis, MN, USA; Fig. 1d). Positive, deliberate coronary vessel identification compared to the angiogram in the operating room is a must. The distal anastomoses are constructed as per routine off-pump practice and initiated only once exposure appears good, which may require several attempts to achieve. Grafting must be meticulous and is followed by assessment of graft flow, reversal of heparin with protamine, placement of chest tubes, and lung reinflation. It is important to check that conduits are not under any tension or kinked upon lung reinflation (Fig. 1e) prior to closure of the thoracotomy wound.

## 7. Patient Safety is First and Foremost

Like with any other operation, the patient’s safety is central to MICS CABG. One should not hesitate to use cardiopulmonary bypass support or even convert to full sternotomy if this allows a higher quality of anastomoses or to manage a problem in a timely fashion. Similarly, patients who are likely to have significant ischemic burden, poor exposure, or a larger heart should be considered early and routinely for cardiopulmonary bypass-assisted MICS CABG (Fig. 1f).

## Conclusions

MICS takes surgical revascularization to a new horizon where patients can achieve superior long-term survival without excessive surgical trauma, avoid excess morbidity, and enjoy faster recovery. The learning curve is long; however, these 7 pillars provide the ground rules for a successful transition to a MICS CABG program.

## References

[1] McGinn JT Jr. Usman S. Lapierre H et al. Minimally invasive coronary artery bypass grafting: dual-center experience in 450 consecutive patients. Circulation 2009; 120(11 Suppl): S78–S84. 19752390 10.1161/CIRCULATIONAHA.108.840041

[2] Ruel M. Shariff MA. Lapierre H et al. Results of the Minimally Invasive Coronary Artery Bypass Grafting Angiographic Patency Study. J Thorac Cardiovasc Surg 2014; 147: 203–209. 24183338 10.1016/j.jtcvs.2013.09.016

[3] Une D. Lapierre H. Sohmer B et al. Can minimally invasive coronary artery bypass grafting be initiated and practiced safely: a learning curve analysis. Innovations 2013; 8: 403–409. 24356429 10.1097/IMI.0000000000000019

[4] Lapierre H. Chan V. Sohmer B et al. Minimally invasive coronary artery bypass grafting via a small thoracotomy versus off-pump: a case-matched study. Eur J Cardiothorac Surg 2011; 40: 804–810. 21393011 10.1016/j.ejcts.2011.01.066

